# Intensive Chemotherapy With or Without Midostaurin in Adults ≥ 60 Years Old With FLT3‐Mutated AML: A FILO‐DATAML‐PETHEMA Real‐World Study

**DOI:** 10.1002/ajh.70233

**Published:** 2026-02-11

**Authors:** Gaspar Aspas Requena, Pau Montesinos, Emilie Bérard, Sarah Bertoli, Rebeca Rodríguez‐Veiga, Celestine Simand, Laura Torres, Pierre Peterlin, Mar Tormo, Rudy Birsen, Juan Miguel Bergua‐Burgues, Emmanuelle Tavernier, Teresa Bernal del Castillo, Martin Carré, Cristina Gil, Adrien Contejean, Eduardo Rodríguez‐Arbolí, Corentin Orvain, Carlos Rodríguez‐Medina, Lauren Veronese, Maria Jose Sayas Lloris, Eric Delabesse, Josefina Serrano, Ariane Mineur, María García‐Fortes, Romain Guieze, Maria Luz Amigo, Arnaud Pigneux, Lorenzo Algarra Algarra, Christian Recher, Pierre‐Yves Dumas, David Martínez‐Cuadrón

**Affiliations:** ^1^ Montpellier University Hospital Service d'Hématologie Clinique Montpellier France; ^2^ University of Montpellier Montpellier France; ^3^ Hospital Universitario y Politécnico La Fe Servicio de Hematología Valencia Spain; ^4^ Department of Clinical Epidemiology and Public Health Toulouse University Hospital Toulouse France; ^5^ CERPOP, Inserm University Toulouse III Paul Sabatier Toulouse France; ^6^ Toulouse University Hospital, Institut Universitaire du Cancer de Toulouse–Oncopole, Service d'Hématologie Université de Toulouse Toulouse France; ^7^ Institut de Cancérologie Strasbourg Europe (ICANS) Département d'Hématologie Strasbourg France; ^8^ Hematology Department Nantes University Hospital Nantes France; ^9^ Hospital Clínico Universitario de Valencia, Servicio de Hematología Biomedical Research Institute INCLIVA Valencia Spain; ^10^ Service d'Hématologie Clinique, Hôpital Cochin–Port Royal (AP‐HP), Institut Cochin Université Paris Cité Paris France; ^11^ Hospital San Pedro de Alcántara Servicio de Hematología Cáceres Spain; ^12^ Centre Hospitalier Universitaire de Saint‐Étienne Service d'Hématologie Clinique et Thérapie Cellulaire Saint‐Étienne France; ^13^ Hospital Universitario Central de Asturias Servicio de Hematología Oviedo Spain; ^14^ Centre Hospitalier Universitaire Grenoble‐Alpes Service d'Hématologie Grenoble France; ^15^ Hospital General Universitario de Alicante Servicio de Hematología Alicante Spain; ^16^ Centre Hospitalier Annecy Genevois Service d'Hématologie Clinique Epagny Metz‐Tessy (Annecy) France; ^17^ Hospital Universitario Virgen del Rocío, Servicio de Hematología, Instituto de Biomedicina de Sevilla (IBiS/CSIC) University of Seville Seville Spain; ^18^ Service d'Hématologie CHU d'Angers Angers France; ^19^ CRCINA, Inserm, Université d'Angers Angers France; ^20^ Hospital Universitario de Gran Canaria Dr. Negrín Servicio de Hematología Las Palmas de Gran Canaria Spain; ^21^ Centre Hospitalier Universitaire de Clermont‐Ferrand Service de Cytogénétique Clermont‐Ferrand France; ^22^ Hospital Universitario Dr. Peset Servicio de Hematología Valencia Spain; ^23^ Toulouse University Hospital Institut Universitaire du Cancer de Toulouse–Oncopole, Laboratoire d'Hématologie Toulouse France; ^24^ Hospital Universitario Reina Sofía, IMIBIC Universidad de Córdoba (UCO), Servicio de Hematología y Hemoterapia Córdoba Spain; ^25^ Centre Hospitalier Universitaire de Bordeaux Service d'Hématologie Clinique et de Thérapie Cellulaire Bordeaux France; ^26^ Hospital Universitario Virgen de la Victoria Servicio de Hematología Málaga Spain; ^27^ Centre Hospitalier Universitaire de Clermont‐Ferrand Service d'Hématologie Clinique et de Thérapie Cellulaire Clermont‐Ferrand France; ^28^ Hospital Morales Meseguer Servicio de Hematología Murcia Spain; ^29^ Hospital General Universitario de Albacete Servicio de Hematología Albacete Spain

**Keywords:** elderly patients, FLT3‐mutated AML, intensive chemotherapy, midostaurin, real‐world data

## Abstract

The addition of midostaurin (MIDO) to intensive chemotherapy (IC) improves survival in younger adults with *FLT3*‐mutated acute myeloid leukemia (AML); however, real‐world data in elderly patients (≥ 60 years) are limited. This large, retrospective, multicenter study from three European registries (PETHEMA, FILO, DATAML) evaluated MIDO+IC (*n* = 194) versus IC alone (*n* = 371) in 565 patients with *FLT3*‐mutated AML aged ≥ 60 years (median age 67.5 years; 35.6% ≥ 70 years). MIDO+IC was associated with lower day‐60 early death (8.2% vs. 21.4%, *p* < 0.0001) and higher composite complete remission (CRc) rates (78.9% vs. 63.1%, *p* < 0.0001). After a median follow‐up of 46.0 months, median overall survival (OS) was 24.2 months for MIDO+IC versus 8.7 months for IC (*p* < 0.0001), with 5‐year OS rates of 40.6% vs. 12.9%, respectively. Event‐free survival (EFS; median 13.5 vs. 4.6 months; 5‐year EFS: 36.0% vs. 10.1%) and relapse‐free survival (RFS; median 20.2 vs. 8.0 months; 5‐year RFS: 45.4% vs. 15.7%) were also significantly improved (both *p* < 0.0001). The 5‐year cumulative incidence of relapse was lower with MIDO+IC (47.8% vs. 67.1%, *p* < 0.001). In multivariate analyses, midostaurin was an independent favorable prognostic factor for CRc (aOR 1.97 [95% CI: 1.29–2.98]), OS (aHR 0.46 [95% CI: 0.36–0.58]), EFS (aHR 0.49 [95% CI: 0.39–0.60]), and RFS (aHR 0.47 [CI: 0.36–0.62]) (all *p* ≤ 0.002). These benefits were confirmed by propensity score matching. This large real‐world study demonstrates that combining midostaurin with IC significantly improves remission rates and survival outcomes in elderly patients with *FLT3*‐mutated AML, supporting its consideration in this population.

## 1 Introduction

*FLT3* mutations are among the most frequent genetic alterations (≈30%) in acute myeloid leukemia (AML), particularly internal tandem duplications (ITD, ≈25%), which are associated with high leukemic burden and poor prognosis; point mutations in the tyrosine kinase domain (TKD, ≈5%) also occur; however, the effect of *FLT3*‐TKD mutations on patient prognosis remains uncertain [1, 2]. *FLT3* is a well‐validated therapeutic target, and midostaurin (MIDO), a type I multikinase inhibitor with activity against both *FLT3*‐ITD and TKD mutations [3], was the first *FLT3* inhibitor to demonstrate a survival benefit when combined with intensive chemotherapy (IC). In the pivotal phase 3 RATIFY trial (CALGB 10603), the addition of MIDO to standard (7 + 3) induction chemotherapy with daunorubicin, high‐dose cytarabine (HDAC) consolidation and one‐year maintenance significantly improved overall survival (OS) and event‐free survival (EFS) in newly diagnosed *FLT3*‐mutated AML patients aged 18 to 59 years [4].

However, RATIFY excluded patients aged ≥ 60 years, leaving a substantial evidence gap in this increasingly prevalent age group. The German‐Austrian AMLSG 16–10 trial, a phase 2 study including patients up to 70 years, based on a historical control, suggested improved outcomes with MIDO in older patients receiving daunorubicin‐based chemotherapy [5]. Yet, real‐world data on the use of MIDO in patients ≥ 60 years remain limited, particularly in those treated with idarubicin‐based regimens, which are frequently used. More recently, observational studies have reported on MIDO use in real‐life cohorts using idarubicin‐based backbones [6, 7, 8], but results remain heterogeneous and inconclusive.

The aim of our study was to assess the real‐world safety and efficacy of combining MIDO with intensive chemotherapy in patients over 60 years of age by compiling a large real‐life series of patients. This retrospective multicenter study aims at bridging this gap by evaluating real‐world outcomes of MIDO therapy in older patients, providing critical insights into its clinical utility and informing future treatment strategies for this population.

## 2 Materials and Methods

### 2.1 Patients

This retrospective, multicenter study included data from adult patients registered in three observational databases: the Spanish PETHEMA (Programa Español de Tratamientos en Hematología) registry (NCT02607059), the French Innovative Leukemia Organization (FILO) registry, and the French Toulouse‐Bordeaux (DATAML) registry. For this analysis, patients were eligible if they were aged ≥ 60 years, had newly diagnosed Acute Myeloid Leukemia (AML) according to the World Health Organization (WHO) 2016 classification [9], harbored a *FLT3* mutation and were treated with intensive chemotherapy. Patients with acute promyelocytic leukemia were excluded. The study period for patient inclusion was from January 1, 2005, to August 31, 2023. The study was conducted in accordance with the Declaration of Helsinki. The protocols of the participating registries were approved by the respective institutional review boards or ethics committees of the participating centers or cooperative groups, and written informed consent for data collection and anonymized research use was obtained from all patients at the time of registration in their respective databases.

### 2.2 Genetic Analysis

*FLT3*‐ITD and ‐TKD mutations and *NPM1* mutations were identified at local participating institutions as part of the standard diagnostic workup at the time of patient inclusion, using either polymerase chain reaction (PCR)‐based assays or next‐generation sequencing (NGS) methods prevalent at the time. The *FLT3*‐ITD allelic ratio (AR) was determined locally and collected; for analytical purposes, it was categorized using a threshold of > 0.5 versus ≤ 0.5. Cytogenetic risk stratification was performed according to the United Kingdom Medical Research Council (MRC) classification [10].

### 2.3 Study Design

**FIGURE 1:**
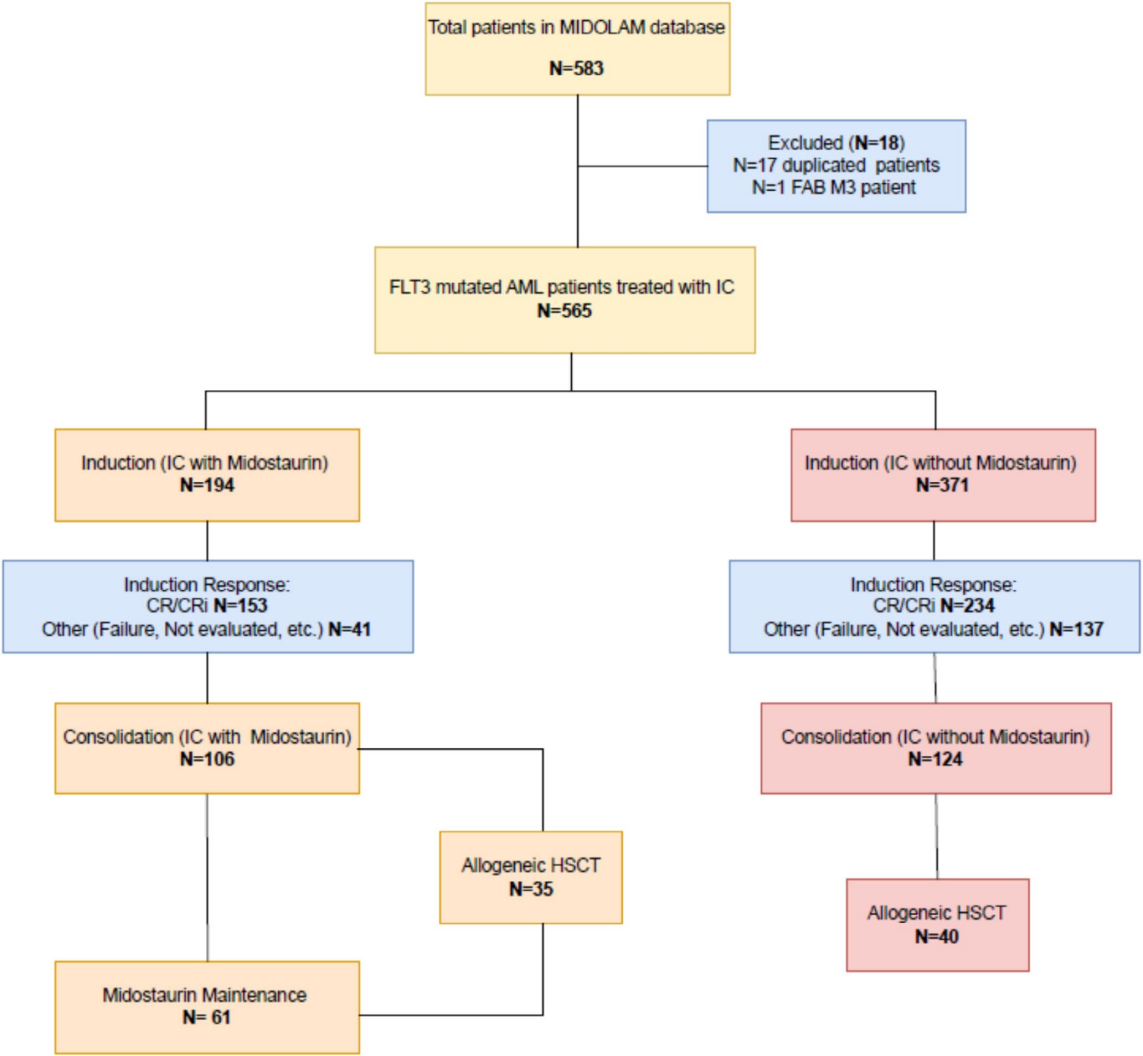
Flow chart diagram. CR/CRi: complete remission/complete remission with incomplete hematologic recovery. HSCT: allogeneic stem cell transplantation. IC: intensive chemotherapy. [Color figure can be viewed at wileyonlinelibrary.com]

This study was designed as a retrospective, multicenter analysis of registry data to evaluate the efficacy of IC with or without MIDO in elderly patients (≥ 60 years) with newly diagnosed *FLT3*‐mutated AML. The overall study design and patient flow are illustrated in the flow chart diagram (Figure 1).

#### 2.3.1 Induction Chemotherapy

Patients received various intensive chemotherapy regimens. The most common first induction course consisted of idarubicin and cytarabine based chemotherapy (7 + 3 regimen) or (5 + 2 regimen) (idarubicin 12 mg/m^2^/day on days 1–3, with cytarabine 100–200 mg/m^2^/day on days 1–7 or idarubicin 8–12 mg/m^2^/day on days 1–2, with cytarabine 100–200 mg/m^2^/day on days 1–5), which in some cases included lomustine [11] (idarubicin 8 mg/m^2^/day on days 1–5, cytarabine 100 mg/m^2^/day on days 1–7, with lomustine 200 mg/m^2^ on day 1) or gemtuzumab ozogamycin (idarubicin 12 mg/m^2^/day on days 1–3, cytarabine 100–200 mg/m^2^/day on days 1–7, with gemtuzumab ozogamicin 3 mg/m^2^ on days 1, 4, and 7). Other patients received daunorubicin and cytarabine based chemotherapy (daunorubicin 60–90 mg/m^2^/day on days 1–3, with cytarabine 100–200 mg/m^2^/day on days 1–7), including CPX‐351 induction regimen (Liposomal daunorubicin 44 mg/m^2^ and cytarabine 100 mg/m^2^ on days 1, 3, and 5). A small proportion of patients received other diverse chemotherapy regimens as detailed in Table S1. For patients in the midostaurin group (IC + MIDO), midostaurin was administered orally at a dose of 50 mg twice daily on days 8 through 21 of the induction cycle.

#### 2.3.2 Consolidation Therapy

Patients achieving CR or CRi after induction were eligible for consolidation therapy. Consolidation regimens included intermediate or high‐dose cytarabine (I/HDAC)‐based chemotherapy or CPX‐351 (HiDAC: cytarabine 3 g/m^2^ twice daily on days 1, 3, and 5; IDAC: cytarabine 1–1.5 g/m^2^ twice daily on days 1, 3, and 5 or CPX‐351: liposomal daunorubicin 29 mg/m^2^ and cytarabine 65 mg/m^2^ on days 1 and 3), less intensive outpatient “mini‐consolidation regimen” [12] (idarubicin 8 mg/m^2^ on day 1, cytarabine 50 mg/m^2^ twice daily IV on days 1 to 5), or autologous stem cell transplantation (SCT). Patients in the IC + MIDO group who achieved remission continued to receive midostaurin 50 mg twice daily on days 8 to 21 during consolidation chemotherapy cycles.

#### 2.3.3 Maintenance Therapy

Following consolidation, an attempt was made to provide midostaurin maintenance monotherapy to responding patients in the IC + MIDO group. Maintenance consisted of midostaurin 50 mg twice daily for up to 12 cycles of 28 days each. However, due to market access restrictions, great heterogeneity was foreseen.

#### 2.3.4 Allogeneic Stem Cell Transplantation (HSCT)

Allogeneic HSCT was considered for eligible patients based on individual risk assessment, donor availability, and institutional guidelines and practices.

### 2.4 Definition of Response Criteria, Survival Endpoints, and Hematologic Recovery

Response to treatment, including complete remission (CR), complete remission with incomplete hematologic recovery (CRi), overall survival (OS), event‐free survival (EFS), relapse‐free survival (RFS), and cumulative incidence of relapse (CIR), was defined as follows: OS was measured from the date of initiation of IC to the date of death from any cause or last follow‐up if alive; EFS was calculated from the date of IC to the first event—defined as failure to achieve CR/CRi, relapse, or death—or last follow‐up if no event occurred; RFS was assessed from the date of CR or CRi to the first event—relapse or death—or last follow‐up if no event occurred, as the date of last follow‐up was considered equivalent to the last disease evaluation in the absence of a documented relapse. Composite complete remission (CRc) was defined as achieving either CR or CRi. Primary refractory AML was defined as failure to achieve CR or CRi after one course of induction chemotherapy. Relapse was defined as ≥ 5% bone marrow blasts, reappearance of blasts in peripheral blood, or development of extramedullary disease after achieving remission. Hematologic recovery criteria are implicitly included within the European LeukemiaNet (ELN) 2022 definitions of CR and CRi [13]. Early death was assessed at day 30 and day 60 post‐induction initiation.

### 2.5 Statistical Analysis

Prior to analysis, data were verified for missing, aberrant, or inconsistent values. After corrections, the database was locked for analysis. Analyses were performed on the locked database. Descriptive statistics were used to summarize patient and treatment characteristics: numbers and frequencies (of non‐missing data) for qualitative variables; and median, interquartile range (IQR; 25th–75th percentiles), and range (min‐max) for quantitative variables.

Survival outcomes (OS, EFS, RFS) were estimated using the Kaplan–Meier method, and curves were compared using the log‐rank test. The median follow‐up was calculated using the reverse Kaplan–Meier technique. Due to differing follow‐up durations between treatment groups, patients in the IC without midostaurin group were censored at 60 months for comparative survival analyses to mitigate potential bias. CIR was estimated using cumulative incidence functions, considering non‐relapse mortality as a competing event, and compared using Gray's test [14].

Comparisons of baseline patient and treatment characteristics between the IC with midostaurin and IC without midostaurin groups were performed using Student's *t*‐test or Mann–Whitney *U* test for continuous variables (based on normality and homoscedasticity) and the *χ* ^2^‐test or Fisher's exact test for categorical variables, as appropriate.

To assess the independent prognostic impact of midostaurin, multivariate Cox proportional hazards models were used for OS, EFS, and RFS, and a multivariate logistic regression model was used for CRc rates. Variables considered for inclusion in the multivariate models were treatment group (midostaurin vs. no midostaurin) together with potential confounding factors: age (≥ 70 vs. < 70 years), sex (male vs. female), ECOG performance status (0–1 vs. ≥ 2), AML status (de novo vs. secondary), baseline white blood cell (WBC) count (≥ 30 × 10^9^/L vs. < 30 × 10^9^/L), cytogenetic risk (favorable/intermediate vs. adverse), *FLT3*‐ITD mutation presence, *FLT3*‐*ITD* ratio (> 0.5 vs. ≤ 0.5), *FLT3*‐*TKD* mutation presence, *NPM1* mutation, and allogeneic HSCT (as a time‐dependent covariate for survival endpoints only). A stepwise selection procedure was applied until only variables significantly and independently associated with the outcome (*p* < 0.05) remained in the final model. The proportional‐hazards assumption for Cox models was checked using “log–log” plots for each covariate. Interactions between significant independent covariates and midostaurin treatment were tested in the final models. None was significant.

As a sensitivity analysis to account for potential baseline imbalances between treatment groups, a propensity score matching (PSM) analysis was performed. A logistic regression model was generated to estimate each patient's propensity score for receiving midostaurin. Covariates included in the propensity score model were age, sex, ECOG performance status, AML status, baseline WBC count, cytogenetic risk, *FLT3*‐ITD mutation, *FLT3*‐ITD ratio, *FLT3*‐TKD mutation, *NPM1* mutation, and French–American–British (FAB) classification. The model's performance was assessed using the Hosmer–Lemeshow statistic and the c‐statistic.

All reported *p*‐values were two‐sided, and a *p*‐value < 0.05 was considered statistically significant. Statistical analyses were performed using STATA version 18.0 (StataCorp LLC, College Station, TX, USA).

## 3 Results

### 3.1 Study Population

A total of 583 patients with newly diagnosed *FLT3*‐mutated AML were initially identified and fulfilled the inclusion criteria. After excluding 17 duplicate patients and 1 patient with a rare acute promyelocytic leukemia transcript, the analyzed cohort comprised 565 patients. The overall study design and patient flow are illustrated in the flow chart diagram (Figure 1). The characteristics of these 565 patients are detailed in Table 1. Among them, 371 (65.7%) received intensive chemotherapy without midostaurin (IC group), collected between January 2005 and June 2017, and 194 (34.3%) received IC combined with midostaurin (IC + MIDO group), collected between June 2017 and August 2023.

**TABLE 1:** Baseline characteristics at AML diagnosis.

	IC *n* = 371 (65.7%)	IC + MIDO *n* = 194 (34.3%)	Total *n* = 565 (100%)	*p*
Age (years) at diagnosis				
Median (IQR)	67.3 (63.6–72.5)	67.6 (63.8–70.9)	67.5 (63.7–71.8)	0.248
Range	60.0–81.4	60.2–77.7	60.0–81.4	
Gender: *n* (%)				
Male	198 (53.4)	97 (50.0)	295 (52.2)	0.446
Female	173 (46.6)	97 (50.0)	270 (47.8)	
ECOG at diagnosis: *n* (%)				
0–1	249 (72.8)	162 (86.6)	411 (77.7)	< 0.001
≥ 2	93 (27.2)	25 (13.4)	118 (22.3)	
WBC at diagnosis (×109/L)				
Median (IQR)	54.9 (18.2–133.2)	25.4 (6.3–96.6)	37.0 (12.8–101)	< 0.001
Range	0.3–483.6	0.5–890.0	0.3–890.0	
BM blast at diagnosis (%)				
Median (IQR)	77.0 (51.0–89.0)	76.0 (54.0–88.0)	77.0 (52.0–88.0)	0.696
Range	0.0–100	3.0–99.0	0.0–100	
AML status: *n* (%)				
De novo	299 (80.8)	156 (81.2)	455 (81.0)	0.899
Secondary AML^a^	71 (19.2)	36 (18.8)	107 (19.0)	
FAB Classification: *n* (%)^b^				
MO	17 (5.3)	10 (6.3)	27 (5.6)	
M1	99 (30.9)	39 (24.5)	138 (28.8)	
M2	46 (14.4)	21 (13.2)	67 (14.0)	0.004
M4	81 (25.3)	25 (15.7)	106 (22.1)	
M5	57 (17.8)	42 (26.4)	99 (20.7)	
M6	1 (0.3)	0 (0.0)	1 (0.2)	
Unclassifiable	19 (5.9)	22 (13.8)	41 (8.6)	
Cytogenetics risk: *n* (%)^c^				
Favorable	4 (1.1)	3 (1.5)	7 (1.3)	0.715
Intermediate	330 (92.7)	182 (93.8)	512 (93.1)	
Adverse	22 (6.2)	9 (4.6)	31 (5.6)	
*FLT3*‐ITD mutation: *n* (%)^d^	329 (88.7)	154 (79.4)	483 (85.5)	0.002
*FLT3*‐TKD mutation: *n* (%)^d^	51 (19.5)	47 (24.2)	98 (21.5)	0.229
*FLT3* AR ITD/wt: *n* (%)				
≤ 50%	123 (45.2)	67 (45.0)	190 (45.1)	0.960
> 50%	149 (54.8)	82 (55.0)	231 (54.9)	
*NPM1* mutation: *n* (%)	214 (58.8)	127 (66.5)	341 (61.4)	0.076

Abbreviations: AML, acute myeloid leukemia; AR, allelic ratio; ECOG, performance status; IC, intensive chemotherapy; IQR, interquartile range; ITD, internal tandem duplication; MIDO, midostaurin; TKD, tyrosine kinase domain; WBC, white blood cells.

^a^ Non‐de novo AML (35 post MDS, 7 post MPN, 48 post CMML, 10 therapy‐related, and 7 others).

^b^ According to French American British classification.

^c^ According to MRC 2010 classification.

^d^ One patient can be included in both subgroups ‐ITD and TKD mutations (16 displayed both mutations).

The median age for the entire cohort was 67.5 years (range: 60.0–81.4 years), with 201 patients (35.6%) aged ≥ 70 years. Males constituted 52.2% of the patients, and 19.0% had secondary AML. Regarding cytogenetic risk according to the MRC classification, the majority of patients (93.1%) belonged to the intermediate‐risk group.

*FLT3*‐ITD mutation was present in 483 patients (85.5%). Significant baseline differences were observed between the IC and IC + MIDO groups (Table 1). The IC group had a higher median white blood cell count (WBC) (54.9 × 10^9^/L [IQR, 18.2–133.2] vs. 25.4 × 10^9^/L [IQR, 6.3–96.6] for IC + MIDO; *p* < 0.001), a greater proportion with ECOG performance status ≥ 2 (27.2% vs. 13.4%; *p* < 0.001), and a higher prevalence of *FLT3*‐ITD mutations (88.7% vs. 79.4%; *p* = 0.002). The prevalence of *FLT3*‐TKD mutations, identified in 98 patients (21.5%) of the cohort, did not significantly differ between groups (19.5% in IC vs. 24.2% in IC + MIDO; *p* = 0.229). *NPM1* mutations, found in 341patients (61.4%), were also similarly distributed (58.8% vs. 66.5%; *p* = 0.076).

### 3.2 Induction Chemotherapy, Response Rates, and Consolidation Therapy

The induction chemotherapy regimens are detailed in Table S1. Predominantly, idarubicin‐based regimens were administered, with idarubicin plus cytarabine (“7 + 3”) given to 273 patients (48.3%), and idarubicin “7 + 3” combined with lomustine (CCNU) to 183 patients (32.4%). Gemtuzumab ozogamicin was added to an idarubicin‐based regimen for 3 patients. Daunorubicin‐based regimens were used in 50 patients (8.9%), which included standard daunorubicin “7 + 3” for 35 (6.2%) and CPX‐351 for 15 patients (2.7%). Others intensive chemotherapy regimens were administered to 21 patients (3.7%).

After excluding 7 patients who died before day 8 of induction (all from the IC group), early death (ED) rates were significantly lower in the IC + MIDO group compared to the IC group. ED by day 30 occurred in 7 IC + MIDO patients (3.6%) versus 59 IC patients (16.2%) (*p* < 0.0001). Similarly, ED by day 60 was 8.2% in the IC + MIDO group versus 21.4% in the IC group (*p* < 0.0001).

Following one cycle of induction chemotherapy, the composite complete remission (CRc) rate, defined as CR plus CRi, was significantly higher in the IC + MIDO group 78.9% compared to 63.1% in the IC group (*p* < 0.0001). Detailed responses are presented in Table 2. The overall response rate (ORR; CRc + partial remission [PR]) was 80.9% for IC + MIDO versus 66.6% for IC. Persistent AML after one cycle of induction chemotherapy was comparable between the IC + MIDO group (13.4%) and the IC group (14.6%). Absence of response evaluation after induction was significantly less frequent in the IC + MIDO group (4.6%) compared to the IC group (18.9%; *p* < 0.0001).

**TABLE 2:** Response to induction chemotherapy.

	IC *n* = 371 (65.7%)	IC + MIDO *n* = 194 (34.3%)	Total *n* = 565 (100%)	*p*
Combined criteria for best response: *n* (%)				
CR	204 (55.0)	138 (71.1)	342 (60.5)	
CRi	30 (8.1)	15 (7.7)	45 (8.0)	
CRc^a^	234 (63.1)	153 (78.9)	387 (68.5)	
PR	13 (3.5)	4 (2.1)	17 (3.0)	< 0.001
ORR^b^	247 (66.6)	157 (80.9)	404 (71.5)	
MLFS	0 (0.0)	2 (1.0)	2 (0.4)	
Failure^c^	54 (14.6)	26 (13.4)	80 (14.2)	
No evaluation	70 (18.9)	9 (4.6)	79 (14.0)	

Abbreviations: CR, complete remission; CRi, complete remission with incomplete hematologic recovery; IC, Intensive chemotherapy; IQR, interquartile range; MIDO, midostaurin; MLFS, morphological leukemia‐free state; ORR, overall response rate; PR, partial remission.

^a^ Composite complete remission (CRc) = CR + CRi.

^b^ Overall response rate (ORR) = CRc + PR.

^c^ Failure = progression + stable disease.

Multivariate logistic regression analysis showed that midostaurin treatment was significantly and independently associated with an increased likelihood of achieving CRc (aOR 1.97, [95% CI: 1.29–2.98], *p* = 0.002). In contrast, age ≥ 70 years (aOR 0.61, [95% CI: 0.42–0.89], *p* = 0.010), ECOG ≥ 2 at diagnosis (aOR 0.64, [95% CI: 0.41–0.99], *p* = 0.047), and adverse cytogenetic risk (aOR 0.39, [95% CI: 0.19–0.83], *p* = 0.015) were each associated with a reduced likelihood of achieving CRc (Table 3).

**TABLE 3:** Logistic regression model for factors independently associated with CR/CRi.

	*n*	Events	aOR	95% CI	*p*
Midostaurin					
No	371	234	1	—	—
Yes	194	153	1.97	1.29–2.98	0.002
ECOG at diagnosis					
0–1	411	297	1	—	—
≥ 2	118	69	0.64	0.41–0.99	0.047
Age at diagnosis					
< 70 years	364	265	1	—	—
≥ 70 years	201	122	0.61	0.42–0.89	0.010
Cytogenetics risk					
Fav/Intermediate	519	362	1	—	—
Adverse	31	15	0.39	0.19–0.83	0.015

Abbreviations: aOR, adjusted odds ratio; CI, confidence interval; CR, complete remission; CRi, complete remission with incomplete hematologic recovery; ECOG, performance status.

Consolidation therapy was administered to 387 patients achieving CRc. I/HDAC regimens were received by 230 patients: 124 (54.9%) in the IC group and 106 (69.3%) in the IC + MIDO group. The median number of I/HDAC cycles was 2 (range: 1–3) in the IC group and 2 (range: 1–4) in the IC + MIDO group; patients in the IC + MIDO group tended to receive a greater number of cycles (*p* = 0.003). Less intensive outpatient mini‐consolidations were administered to 105 patients: 74 (81.7%) in the IC group and 31 (66.0%) in the IC + MIDO group. The median number of mini‐consolidation cycles was 4 (range: 1–7) in the IC group and 5 (range: 1–7) in the IC + MIDO group, with no significant difference observed (*p* = 0.272). Autologous stem cell transplantation was performed in 18 CRc patients (4.7%). Allogeneic HSCT was performed in 35 patients (18.0%) in the IC + MIDO group compared to 40 (10.8%) in the IC group (*p* = 0.015). Patients in the IC + MIDO arm achieving remission continued midostaurin during consolidation cycles including mini‐consolidations.

### 3.3 Maintenance

A total of 61 patients initiated maintenance therapy with midostaurin. Among the 106 patients who started consolidation chemotherapy, 59 (55.7%) subsequently proceeded to midostaurin maintenance. Additionally, of the 35 patients who underwent allogeneic HSCT, 6 (17.1%) received post‐transplant maintenance with midostaurin.

## 4 Outcomes

To mitigate potential bias from differing follow‐up durations between treatment groups, patients in the IC group were censored at 60 months for comparative survival analyses. The median follow‐up for the entire cohort after this censoring was 46.0 months (IQR, 31.5–60.0 months).

**FIGURE 2:**
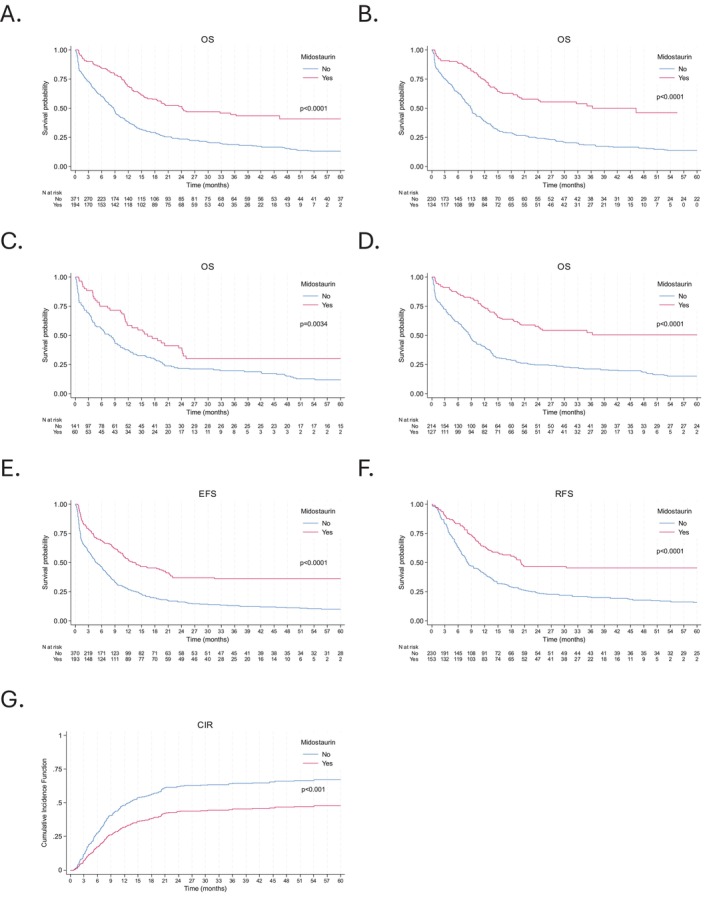
Outcomes among patients with newly diagnosed *FLT3* mutated acute myeloid leukemia according to MIDO treatment: (A) overall survival among the whole cohort (*n* = 565), (B) overall survival among patients < 70 (*n* = 364), (C) overall survival among patients ≥ 70 (*n* = 201), (D) overall survival among patients with *NPM1* mutation (*n* = 341), (E) event‐free survival among the whole cohort (*n* = 563, 2 patients excluded because of missing date of primary induction failure), (F) relapse‐free survival among the whole cohort (*n* = 383, 4 patients excluded because of missing date of CR/CRi), and (G) cumulative incidence of relapse. [Color figure can be viewed at wileyonlinelibrary.com]

Overall survival (OS) was significantly improved in the IC + MIDO group compared to the IC group (*p* < 0.0001; Figure 2A). The median OS was 24.2 months (IQR, 10.5‐NR) for the IC + MIDO group versus 8.7 months (IQR, 2.4–21.7) for the IC group. One‐year, 3‐year, and 5‐year OS rates for the IC + MIDO group were 69.0%, 44.6%, and 40.6%, respectively, compared to 38.1%, 18.9%, and 12.9% for the IC group.

In the subgroup of patients < 70 years (*n* = 364), median OS was 36.4 months (IQR, 11.8‐NR) with IC + MIDO versus 8.9 months (IQR, 3.1–23.5) with IC (*p* < 0.0001; Figure 2B). For patients aged ≥ 70 years (*n* = 201), median OS was 16.3 months (IQR, 5.6‐NR) with IC + MIDO versus 7.8 months (IQR, 1.7–20.1) with IC (*p* = 0.0034; Figure 2C). Among *NPM1*‐mutated patients (*n* = 341), median OS was not reached (IQR, 11.4‐NR) with IC + MIDO versus 8.6 months (IQR, 2.4–23.2) with IC (*p* < 0.0001; Figure 2D).

A Cox proportional hazards model for OS (Table 4) showed that ECOG performance status ≥ 2 (adjusted hazard ratio [aHR] 1.36, 95% CI: 1.07–1.72, *p* = 0.010), secondary AML (aHR 1.37, 95% CI: 1.08–1.75, *p* = 0.010), baseline WBC count ≥ 30 × 10^9^/L (aHR 1.27, 95% CI: 1.03–1.56, *p* = 0.028), and *FLT3*‐ITD allelic ratio > 0.5 (aHR 1.45, 95% CI: 1.15–1.83, *p* = 0.002) were independently associated with shorter OS. Conversely, treatment with midostaurin (IC + MIDO) was independently associated with longer OS (aHR 0.46, 95% CI: 0.36–0.58, *p* < 0.001).

**TABLE 4:** Cox model for factors independently associated with OS.

	*n*	Events	aHR	95% CI	*p*
Midostaurin					
No	371	318	1	—	—
Yes	194	95	0.46	0.36–0.58	< 0.001
AML status					
De novo	455	325	1	—	—
Secondary AML^a^	107	86	1.37	1.08–1.75	0.010
ECOG at diagnosis					
0–1	411	282	1	—	—
≥ 2	118	100	1.36	1.07–1.72	0.010
WBC at diagnosis					
< 30.10^9^/L	232	151	1	—	—
≥ 30.10^9^/L	331	260	1.27	1.03–1.56	0.028
FLT3 AR ITD/wt					
≤ 50%	190	130	1	—	—
> 50%	231	180	1.45	1.15–1.83	0.002

Abbreviations: aHR, adjusted hazard ratio; AML, acute myeloid leukemia; AR, allelic ratio; CI, confidence interval; ECOG, performance status.

^a^ Non‐de novo AML.

Event‐free survival (EFS) was significantly longer with IC + MIDO (Figure 2E); median EFS was 13.5 months (IQR, 3.8‐NR) versus 4.6 months (IQR, 1.1–13.8) with IC (*p* < 0.0001). The 1‐year, 3‐year, and 5‐year EFS rates for the IC + MIDO group were 52.5%, 36.0%, and 36.0%, respectively, compared to 27.0%, 13.2%, and 10.1% for the IC group. EFS outcomes for *NPM1*‐mutated AML patients are presented in Figure S1A. For patients < 70 years and ≥ 70 years, EFS outcomes are shown in Figures S2A and S3A, respectively.

Relapse‐free survival (RFS) was also significantly improved in the IC + MIDO group (Figure 2F), with a median RFS of 20.2 months (IQR, 8.5‐NR) compared to 8.0 months (IQR, 4.0–22.5) for the IC group (*p* < 0.0001). The 1‐year, 3‐years, and 5‐years RFS rates for the IC + MIDO group were 63.4%, 45.4%, and 45.4%, respectively, compared to 40.0%, 20.4%, and 15.7% for the IC group. RFS outcomes for *NPM1*‐mutated patients are presented in Figure S1B. For patients < 70 years and ≥ 70 years, RFS outcomes are shown in Figures S2B and S3B, respectively. Multivariate analyses for EFS and RFS (Tables S2 and S3) indicated that midostaurin was significantly and independently associated with improved outcomes for both.

The cumulative incidence of relapse (CIR) was significantly lower in the IC + MIDO group compared to the IC group (*p* < 0.001; Figure 2G). At 1 year, 3 years, and 5 years, CIR for the IC + MIDO group was 32.2%, 45.3%, and 47.8%, respectively, versus 48.5%, 64.4%, and 67.1% for the IC group. CIR for *NPM1*‐mutated patients is presented in Figure S1C. For patients < 70 years and ≥ 70 years, CIR outcomes are shown in Figures S2C and S3C, respectively. Sensitivity analyses censoring patients at the time of allogeneic HSCT yielded similar results for RFS and CIR (Figure S4).

To assess the potential impact of the “period effect” and improvements in supportive care over the long study duration, another sensitivity analysis has been performed by stratifying the IC group into an “Early Cohort” (2005–2017, *n* = 281) and a “Late Cohort” (2018–2023, *n* = 90). The year 2017 was selected as the cut‐off as it represents the median year of inclusion, dividing the total study population into two numerically balanced groups (50.8% vs. 49.2%). We observed no significant improvement in OS for patients treated with IC alone in the recent period compared to the early period (median OS: 6.5 vs. 8.8 months, respectively; *p* = 0.2755; Figure S5A). Furthermore, the survival benefit of the IC + MIDO group remained statistically significant when compared exclusively to the contemporaneous “Late Control Cohort” (*p* < 0.0001) (Figure S5B).

### 4.1 Sensitivity Analysis Using Propensity Score Matching

To further account for potential baseline differences between the IC and IC + MIDO groups, a propensity score matching (PSM) analysis was performed. A multivariate logistic regression model was generated to estimate each patient's propensity score for receiving midostaurin. Covariates included in this model were age, sex, ECOG performance status, AML status, WBC count, cytogenetic risk, *FLT3*‐ITD mutation, *FLT3*‐ITD allelic ratio, *FLT3*‐TKD mutation, *NPM1* co‐mutation, and FAB classification [15]. The model's performance was assessed using the Hosmer–Lemeshow *χ* ^2^ statistic (*p* = 0.244) and the c‐statistic (0.72, 95% CI: 0.68–0.77). Prior to matching, the mean propensity score was 0.361 (±0.190) in the IC group (*N* = 260 with complete data for PSM) and 0.511 (±0.160) in the IC + MIDO group (*N* = 192 with complete data for PSM). Using these scores, 118 patients receiving midostaurin were matched on a 1:1 basis with 118 patients not receiving midostaurin (104 with a precision of 0.0001, 0 with a precision of 0.001, 32 with a precision of 0.01 and 100 with a precision of 0.1). In the matched sample of 236 patients, mean propensity scores were well balanced between the IC + MIDO group (0.465 ± 0.143) and the IC group (0.465 ± 0.144). Outcomes (CRc, OS, EFS, and RFS) were then compared between these matched groups.

**FIGURE 3:**
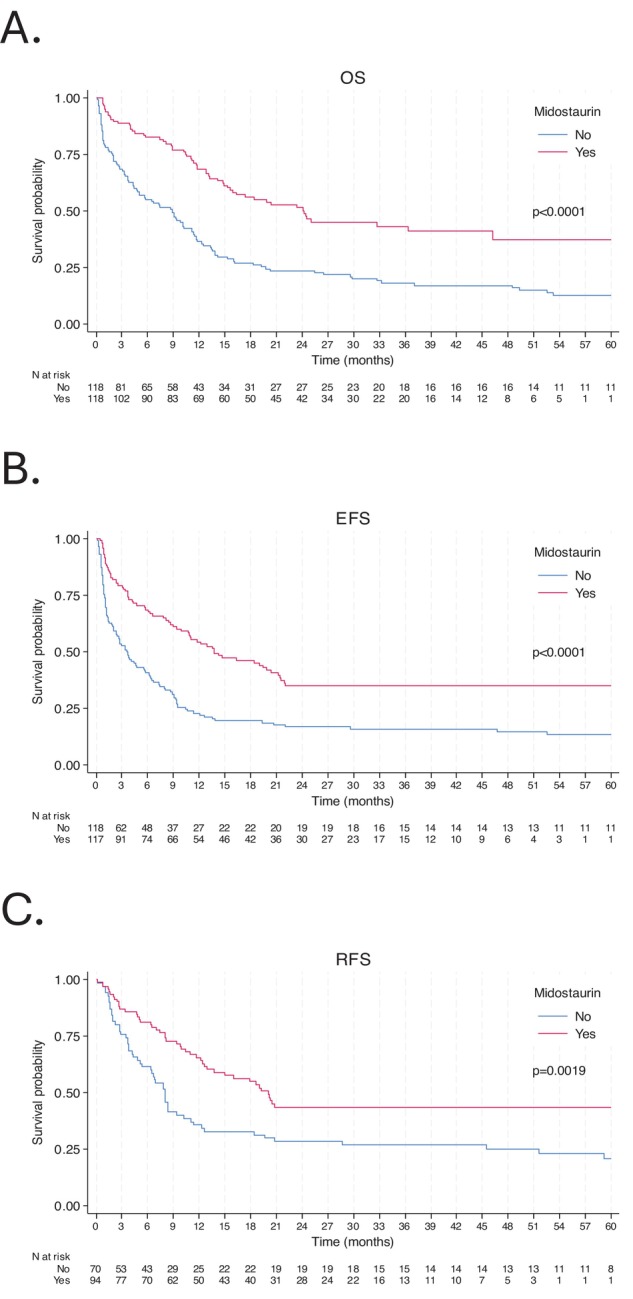
Outcomes among the 236 matched patients with newly diagnosed *FLT3* mutated acute myeloid leukemia: (A) overall survival, (B) event‐free survival, and (C) relapse‐free survival. [Color figure can be viewed at wileyonlinelibrary.com]

In the propensity score‐matched cohort (*N* = 236), the CRc rate was significantly higher in the IC + MIDO group (79.7%, 94/118) compared to the IC group (60.2%, 71/118; *p* = 0.001). Treatment with midostaurin in the matched cohort also resulted in significantly improved survival outcomes (Figure 3). Median OS was 24.2 months (IQR, 10.5‐NR) for the IC + MIDO group versus 8.9 months (IQR, 1.9–19.7) for the IC group (*p* < 0.0001). Median EFS was 13.8 months (IQR, 3.7‐NR) for IC + MIDO versus 3.5 months (IQR, 1.0–10.4) for IC (*p* < 0.0001). Similarly, median RFS was 20.1 months (IQR, 8.0‐NR) for IC + MIDO compared to 8.0 months (IQR, 3.5–45.4) for IC (*p* = 0.0019).

## 5 Discussion

Our real‐world study on a large cohort of elderly patients (≥ 60 years) with newly diagnosed *FLT3*‐mutated AML suggests that the addition of midostaurin to intensive chemotherapy is associated with improved OS compared to IC alone. This finding, from a retrospective analysis, aligns with the survival benefits demonstrated for midostaurin in younger patients (< 60 years) in the pivotal RATIFY trial [4] and extends observations from the AMLSG 16–10 clinical trial [5] and the Sierra et al. phase 3b study [8], which included older patients (≥ 60 years). Although the primary endpoint of the Sierra et al. study was safety and survival data were not systematically collected post‐treatment, both this study and the AMLSG trial supported the feasibility and activity of midostaurin combinations in older adults.

The baseline characteristics of our cohort reflect a challenging, older population, with a median age of 67.5 years and 35.6% of patients aged ≥ 70 years. This contrasts with the RATIFY trial, which was limited to patients < 60 years. In comparison, our cohort is representative of older patients, aligning more closely with the 61–70‐year‐old subset of the AMLSG 16–10 trial and the population studied by Sierra et al., in which 47.2% of patients were over 60 years (despite a median age of 59 years) and only a limited number of patients aged ≥ 70 years. The inclusion of a significant number of patients over 70 years of age in our real‐world setting provides valuable insights into a group often underrepresented in clinical trials. However, we acknowledge notable baseline differences between our IC and IC + MIDO groups, particularly regarding *FLT3*‐ITD mutation prevalence, ECOG performance status, and WBC counts, which necessitated adjustments in our analyses, including propensity score matching, to mitigate confusion bias.

The CRc rate of 78.9% observed in our IC + MIDO group is comparable and encouraging when contextualized with other studies. For instance, the AMLSG 16–10 trial reported a CR + CRi rate of 77.9% in patients aged 61–70 years treated with midostaurin plus chemotherapy. Similarly, the Sierra et al. phase 3b study showed an overall CR + CRi of 80.7%, with 82.5% in patients > 60 to ≤ 70 years and 64.1% in those > 70 years. The CR rate in the RATIFY midostaurin arm was 58.9% (CR + CRi not explicitly reported as a combined primary endpoint). Furthermore, we showed that patients treated with midostaurin experienced a 20% reduction in the 5‐year cumulative incidence of relapse, a clinically relevant observation consistent with later analyses of the RATIFY study.

A distinctive feature of our study is the predominant use of idarubicin‐based induction regimens, reflecting common real‐world practice in many European centers. This contrasts with the RATIFY trial, which exclusively used daunorubicin (60 mg/m^2^) in its standard “7 + 3” backbone, and the AMLSG 16–10 trial, which also employed daunorubicin‐based chemotherapy. The Sierra et al. study, however, allowed for both daunorubicin (60–90 mg/m^2^) and idarubicin (12 mg/m^2^), with 55.1% of patients receiving idarubicin and reported similar CR + CRi rates irrespective of the anthracycline used. Our results with various regimens predominantly based on idarubicin contribute to the evidence supporting midostaurin's efficacy with different anthracycline partners.

Regarding post‐remission therapy, our data did not show a clear impact of HSCT on outcomes in this elderly population, which warrants careful interpretation. While the allo‐HSCT rates in our cohort (18.0% in the IC + MIDO group and 10.8% in the IC group) are lower than those reported in trials focusing on younger adults (e.g., 57% in RATIFY and 72.4% of remitters in AMLSG 16–10), they reflect the reality of treating an elderly population (≥ 60 years). In fact, these rates are consistent with benchmarks from comparable large cohorts of older AML patients. For instance, the randomized FILO LAM‐SA 2007 trial reported an overall allo‐HSCT rate of only 6.8% in fit elderly patients, and the PETHEMA registry analysis reported a rate of 7% for patients ≥ 60 years [16, 17]. Moreover, in the recent multicenter phase II DEXAML‐02 trial in older AML patients (≥ 61 years), reported an allo‐HSCT rate of 20%, providing a contemporary benchmark for intensively treated older patients [18]. In this context, the rates observed in our study suggest a proactive approach to transplantation compared to historical controls.

The retrospective nature of our analysis is a primary limitation, introducing potential selection bias. Moreover, despite statistical adjustments, differences in baseline characteristics (e.g., poorer ECOG scores and higher WBC counts in the IC group, and a higher prevalence of *FLT3*‐ITD in that group before matching) could have introduced potential confounders. The extended accrual period may also encompass evolving standards of care and HSCT guidelines, which likely influenced outcomes. Furthermore, the lack of systematic *NPM1* mutation‐based minimal residual disease (MRD) assessment is a limitation, given its prognostic significance. Nevertheless, the most relevant finding remains the median OS at 24.2 months observed in the IC + MIDO group, independently of cross‐arm comparisons.

Looking forward, prospective studies are crucial, particularly to define the role of maintenance therapy with FLT3 inhibitors in this older population. Midostaurin maintenance was explored in RATIFY, AMLSG 16–10, and Sierra et al., but its optimal use, duration, and benefit, especially post‐HSCT in older patients, require further investigation. This is particularly relevant in the context of other maintenance options like oral azacitidine (CC‐486) and emerging, more potent FLT3 inhibitors such as quizartinib, whose safety and efficacy in combination or as maintenance in older, less fit patients with FLT3‐mutated AML warrant dedicated trials. However, due to market access restrictions across participating centers, the use of midostaurin maintenance therapy was highly heterogeneous. Consequently, this aspect was not systematically studied nor consistently captured in the registry database. Further prospective studies specifically addressing the role and optimal conditions of maintenance therapy with midostaurin in older patients with FLT3‐mutated AML are warranted to clarify its potential benefit in this population.

An important limitation of our study is the inherent bias associated with historical comparisons, as evolving supportive care and transplant practices over time could potentially influence outcomes. However, in our sensitivity analysis comparing outcomes within the control group across two distinct time periods (2005–2017 vs. 2018–2023), we observed superimposable survival curves. This suggests that the “era effect” alone does not appear to have significantly improved OS in older patients treated exclusively with intensive chemotherapy. This finding, together with the benefit maintained by the midostaurin combination when compared specifically against the contemporaneous late control cohort, supports the hypothesis that the observed survival advantage is primarily attributable to the addition of the FLT3 inhibitor rather than to secular trends in clinical management. Nevertheless, we interpret these data with caution, acknowledging baseline imbalances between groups—such as *FLT3*‐ITD frequency and WBC counts—which necessitated the statistical adjustments and propensity score matching performed to substantiate the benefit of midostaurin.

In conclusion, despite the inherent limitations of a retrospective registry‐based comparison, our findings suggest that the combination of midostaurin with intensive chemotherapy is feasible and associated with very good response rates and encouraging survival outcomes in a difficult‐to‐treat, real‐world population of elderly patients with *FLT3*‐mutated AML. These results complement data from prospective trials and support the consideration of this combination for fit older adults.

## Conflicts of Interest

The authors declare no conflicts of interest.

## Supporting information


**Figure S1:** NPM1 patients. (A) Event‐free survival (*n* = 339), (B) Relapse‐free survival (*n* = 250), and (C) Cumulative incidence of relapse.

**Figure S2:** < 70 years old patients. (A) Event‐free survival (*n* = 362), (B) Relapse‐free survival (*n* = 261), and (C) Cumulative incidence of relapse.

**Figure S3:** ≥ 70 years old patients. (A) Event‐free survival (*n* = 201), (B) Relapse‐free survival (*n* = 122), and (C) Cumulative incidence of relapse.

**Figure S4:** Sensitivity analysis after censuring patients at allogeneic HSCT: (A) Relapse‐free survival (*n* = 383) and (B) Cumulative incidence of relapse.

**Figure S5:** Sensitivity analysis evaluating the impact of the treatment period on overall survival. (A) Kaplan–Meier OS curves comparing the historical Early Control Cohort (2005–2017) versus the Late Control Cohort (2018–2023) within the intensive chemotherapy (IC) group. (B) Kaplan–Meier OS curves comparing the IC + Midostaurin group versus the contemporaneous Late Control Cohort (2018–2023).

**Table S1:** Description of induction chemotherapy regimens.

**Table S2:** Cox model for factors independently associated with EFS.

**Table S3:** Cox model for factors independently associated with RFS.

## Data Availability

The data that support the findings of this study are available on request from the corresponding author. The data are not publicly available due to privacy or ethical restrictions.
