# Supplementary material for: Intensive Chemotherapy With or Without Midostaurin in Adults ≥ 60 Years Old With FLT3‐Mutated AML: A FILO‐DATAML‐PETHEMA Real‐World Study
Source: Am J Hematol. 2026 Feb 11;101(5):949–60. doi: 10.1002/ajh.70233 (PMC13055135; doi:10.1002/ajh.70233)
Supplement: Supplementary file 1 — Figure S1: NPM1 patients. (A) Event‐free survival (n = 339), (B) Relapse‐free survival (n = 250), and (C) Cumulative incidence of relapse. [file AJH-101-949-s003.pptx]

## Slide 1
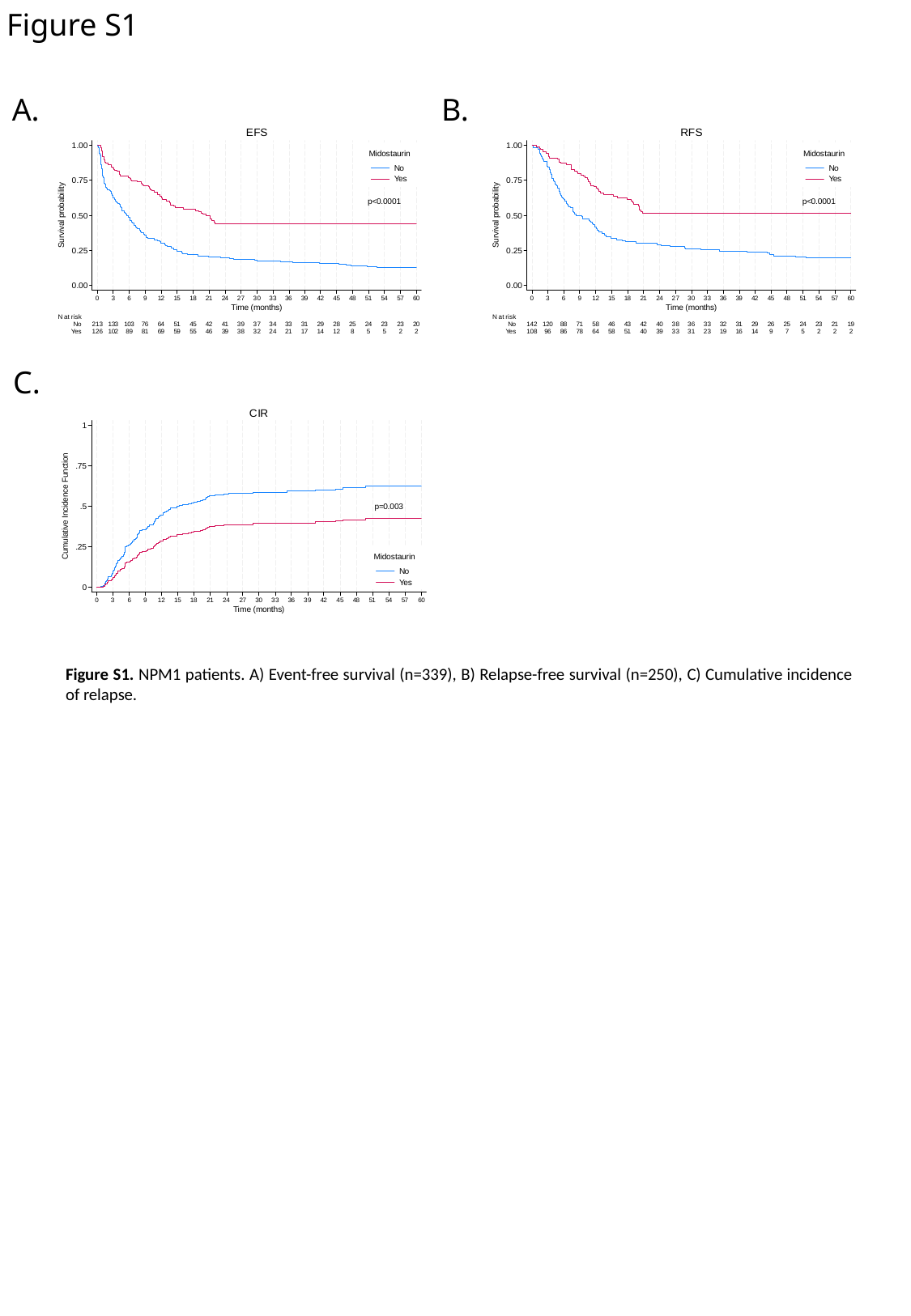

Figure S1
A.
B.
C.
Figure S1. NPM1 patients. A) Event-free survival (n=339), B) Relapse-free survival (n=250), C) Cumulative incidence of relapse.
