# Supplementary material for: Intensive Chemotherapy With or Without Midostaurin in Adults ≥ 60 Years Old With FLT3‐Mutated AML: A FILO‐DATAML‐PETHEMA Real‐World Study
Source: Am J Hematol. 2026 Feb 11;101(5):949–60. doi: 10.1002/ajh.70233 (PMC13055135; doi:10.1002/ajh.70233)
Supplement: Supplementary file 3 — Figure S3: ≥ 70 years old patients. (A) Event‐free survival (n = 201), (B) Relapse‐free survival (n = 122), and (C) Cumulative incidence of relapse. [file AJH-101-949-s002.pptx]

## Slide 1
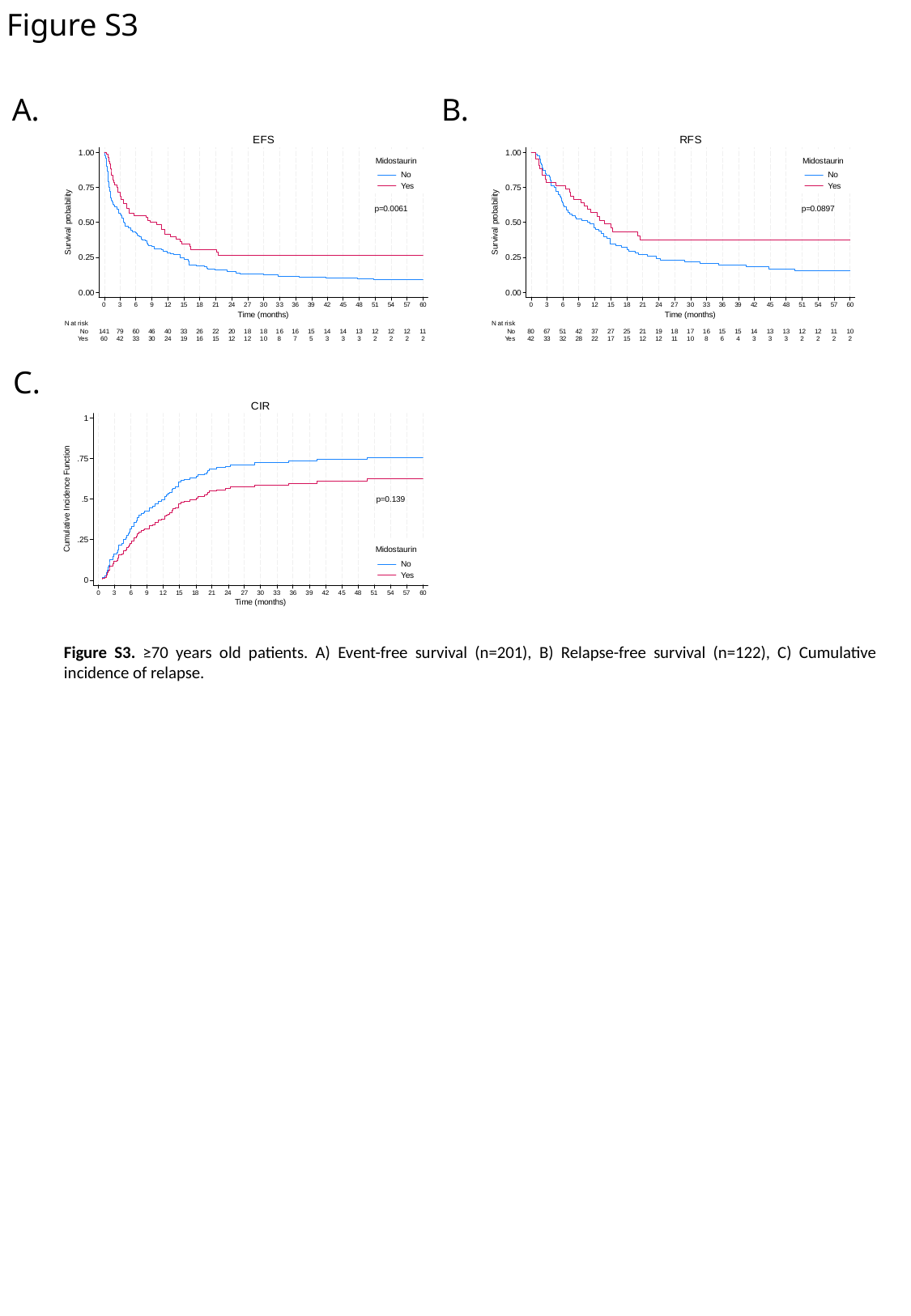

Figure S3
A.
B.
C.
Figure S3. ≥70 years old patients. A) Event-free survival (n=201), B) Relapse-free survival (n=122), C) Cumulative incidence of relapse.
