# Supplementary material for: Intensive Chemotherapy With or Without Midostaurin in Adults ≥ 60 Years Old With FLT3‐Mutated AML: A FILO‐DATAML‐PETHEMA Real‐World Study
Source: Am J Hematol. 2026 Feb 11;101(5):949–60. doi: 10.1002/ajh.70233 (PMC13055135; doi:10.1002/ajh.70233)
Supplement: Supplementary file 4 — Figure S4: Sensitivity analysis after censuring patients at allogeneic HSCT: (A) Relapse‐free survival (n = 383) and (B) Cumulative incidence of relapse. [file AJH-101-949-s008.pptx]

## Slide 1
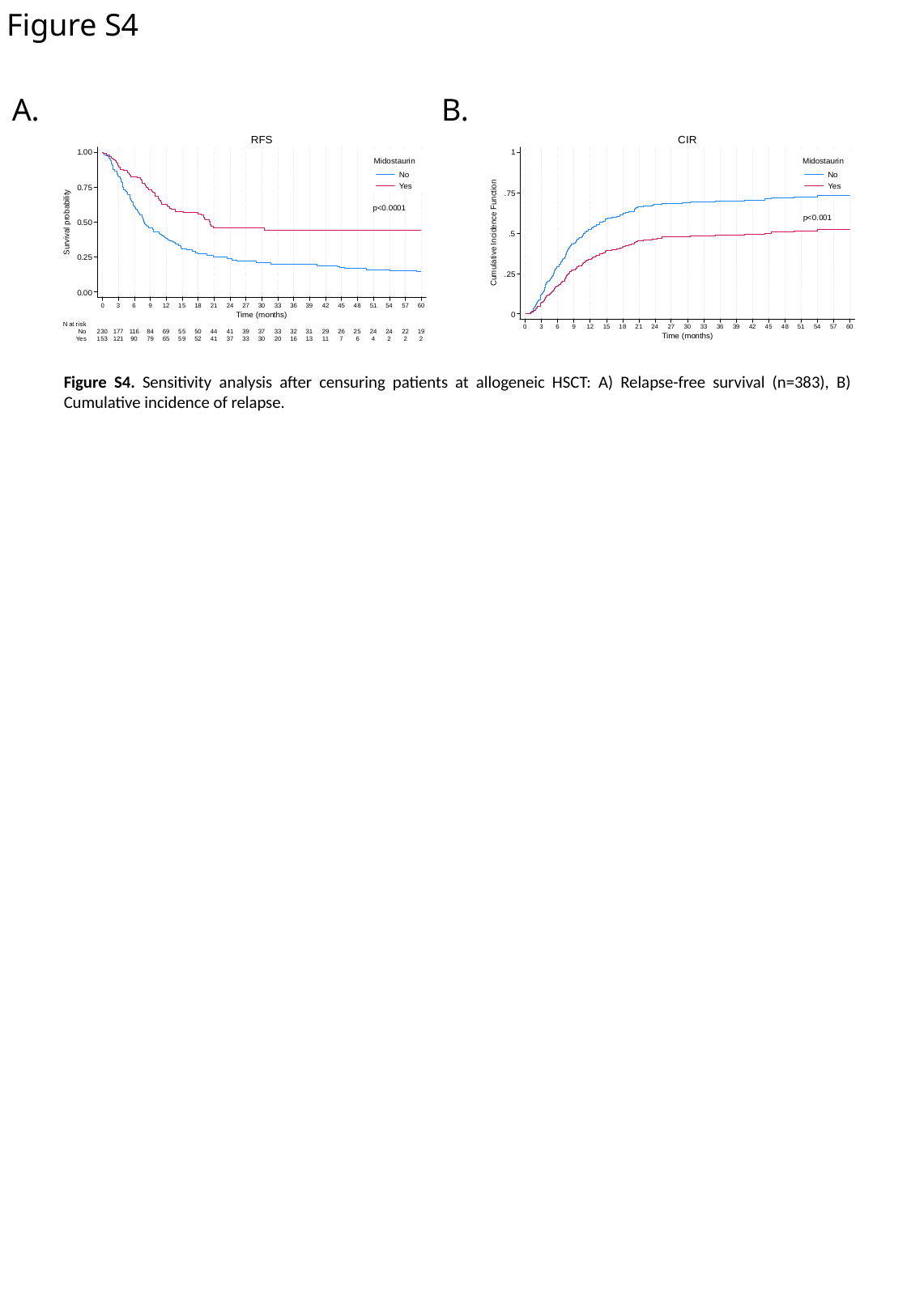

Figure S4
A.
B.
Figure S4. Sensitivity analysis after censuring patients at allogeneic HSCT: A) Relapse-free survival (n=383), B) Cumulative incidence of relapse.
