# Supplementary material for: Intensive Chemotherapy With or Without Midostaurin in Adults ≥ 60 Years Old With FLT3‐Mutated AML: A FILO‐DATAML‐PETHEMA Real‐World Study
Source: Am J Hematol. 2026 Feb 11;101(5):949–60. doi: 10.1002/ajh.70233 (PMC13055135; doi:10.1002/ajh.70233)
Supplement: Supplementary file 5 — Figure S5: Sensitivity analysis evaluating the impact of the treatment period on overall survival. (A) Kaplan–Meier OS curves comparing the historical Early Control Cohort (2005–2017) versus the Late Control Cohort (2018–2023) within the intensive chemotherapy (IC) group. (B) Kaplan–Meier OS curves comparing the IC + Midostaurin group versus the contemporaneous Late Control Cohort (2018–2023). [file AJH-101-949-s005.pptx]

## Slide 1
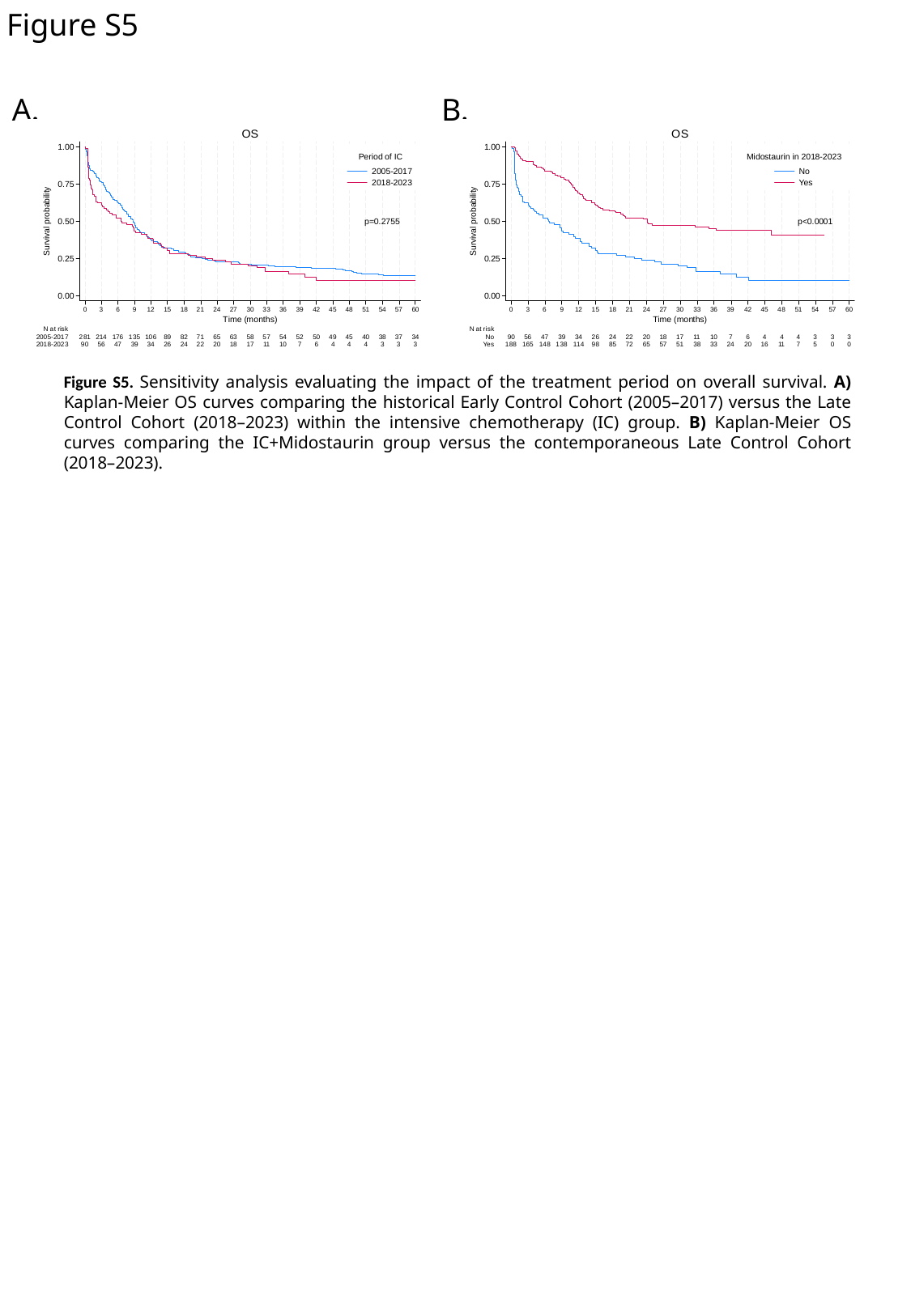

Figure S5
A.
B.
Figure S5. Sensitivity analysis evaluating the impact of the treatment period on overall survival. A) Kaplan-Meier OS curves comparing the historical Early Control Cohort (2005–2017) versus the Late Control Cohort (2018–2023) within the intensive chemotherapy (IC) group. B) Kaplan-Meier OS curves comparing the IC+Midostaurin group versus the contemporaneous Late Control Cohort (2018–2023).
