# Supplementary material for: Intensive Chemotherapy With or Without Midostaurin in Adults ≥ 60 Years Old With FLT3‐Mutated AML: A FILO‐DATAML‐PETHEMA Real‐World Study
Source: Am J Hematol. 2026 Feb 11;101(5):949–60. doi: 10.1002/ajh.70233 (PMC13055135; doi:10.1002/ajh.70233)
Supplement: Supplementary file 6 — Table S1: Description of induction chemotherapy regimens. [file AJH-101-949-s001.docx]

**Table S1. Description of induction chemotherapy regimens**

|  | **IC**  **n = 371 (65.7%)** | **IC+MIDO**  **n = 194 (34.3%)** | **Total**  **n = 565 (100%)** |
| --- | --- | --- | --- |
| Daunorubicin 7+3^a^  CPX-351^b^ | 16 (4.3)  7 (1.9) | 19 (9.8)  8 (4.1) | 35 (6.2)  15 (2.7) |
| Idarubicin 7+3^c^  Idarubicin 5+2^d^  Idarubicin 7+3+CCNU^e^  Idarubicin 7+3+GO^f^ | 166 (44.7)  34 (9.2)  124 (33.4)  3 (0.8) | 107 (55.2)  1 (0.5)  59 (30.4)  0 | 273 (48.3)  35 (6.2)  183 (32.4)  3 (0.5) |
| FLAG-Ida^g^  ICE^h^  MTZ-AraC^i^  Others | 4 (1.1)  8 (2.2)  3 (0.8)  6 (1.6) | 0  0  0  0 | 4 (0.7)  8 (1.4)  3 (0.5)  6 (1.1) |

^a:^ Daunorubicin 60–90 mg/m²/day on days 1–3, with cytarabine 100–200 mg/m²/day on days 1–7

^b:^ Liposomal daunorubicin 44 mg/m² and cytarabine 100 mg/m² on days 1, 3, and 5

^c:^ Idarubicin 12 mg/m²/day on days 1–3, with cytarabine 100–200 mg/m²/day on days 1–7

^d:^ Idarubicin 8–12 mg/m²/day on days 1–2, with cytarabine 100–200 mg/m²/day on days 1–5

^e:^ Idarubicin 8 mg/m²/day on days 1–5, cytarabine 100 mg/m²/day on days 1–7, with lomustine 200 mg/m² on day 1

^f:^ Idarubicin 12 mg/m²/day on days 1–3, cytarabine 100–200 mg/m²/day on days 1–7, with gemtuzumab ozogamicin 3 mg/m² on days 1, 4, and 7

^g:^ Fludarabine 30 mg/m²/day on days 2–5, cytarabine 2 g/m² every 12 h on days 2–6, with idarubicin 12 mg/m²/day on days 4–6

^h:^ Idarubicin 10 mg/m² on days 1, 3, and 5, cytarabine 100 mg/m²/day on days 1–7, with etoposide 100 mg/m²/day on days 1–3

^i:^ Mitoxantrone 8 mg/m²/day on days 1–5, cytarabine 1 g/m²/day on days 1–5, with etoposide 100 mg/m²/day on days 1–5
