# Supplementary material for: Intensive Chemotherapy With or Without Midostaurin in Adults ≥ 60 Years Old With FLT3‐Mutated AML: A FILO‐DATAML‐PETHEMA Real‐World Study
Source: Am J Hematol. 2026 Feb 11;101(5):949–60. doi: 10.1002/ajh.70233 (PMC13055135; doi:10.1002/ajh.70233)
Supplement: Supplementary file 7 — Table S2: Cox model for factors independently associated with EFS. [file AJH-101-949-s006.docx]

**Table S2. Cox model for factors independently associated with EFS**

|  | **n** | **Events** | **aHR** | **95% CI** | **P value** |
| --- | --- | --- | --- | --- | --- |
| **Midostaurin**  No  Yes | 370  193 | 330  113 | 1  0.49 | -  0.39-0.60 | -  <0.001 |
| **AML status**  *De novo*  Secondary AML ^a^ | 454  106 | 351  90 | 1  1.33 | -  1.05–1.68 | -  0.017 |
| **ECOG at diagnosis**  0–1  ≥ 2 | 410  117 | 308  104 | 1  1.31 | -  1.04–1.64 | -  0.021 |
| ***FLT3* ratio ITD/wt**  ≤50%  > 50% | 188  231 | 141  188 | 1  1.37 | -  1.10-1.71 | -  0.005 |

aHR, adjusted hazard ratio; CI, confidence interval; AML, acute myeloid leukemia; ECOG, performance status.

^a^ non-*de novo* AML
