# Supplementary material for: Intensive Chemotherapy With or Without Midostaurin in Adults ≥ 60 Years Old With FLT3‐Mutated AML: A FILO‐DATAML‐PETHEMA Real‐World Study
Source: Am J Hematol. 2026 Feb 11;101(5):949–60. doi: 10.1002/ajh.70233 (PMC13055135; doi:10.1002/ajh.70233)
Supplement: Supplementary file 8 — Table S3: Cox model for factors independently associated with RFS. [file AJH-101-949-s004.docx]

**Table S3. Cox model for factors independently associated with RFS**

|  | **n** | **Events** | **aHR** | **95% CI** | **P value** |
| --- | --- | --- | --- | --- | --- |
| **Midostaurin**  No  Yes | 230  153 | 191  73 | 1  0.47 | -  0.36-0.62 | -  <0.001 |
| **AML status**  *De novo*  Secondary AML ^a^ | 310  71 | 208  55 | 1  1.64 | -  1.21–2.21 | -  0.001 |
| ***FLT3* ratio ITD/wt**  ≤50%  > 50% | 137  155 | 91  112 | 1  1.45 | -  1.10-1.92 | -  0.009 |

aHR, adjusted hazard ratio; CI, confidence interval; AML, acute myeloid leukemia; ECOG, performance status.

^a^ non-*de novo* AML
